# Trial Factors Associated With Completion of Clinical Trials Evaluating AI: Retrospective Case-Control Study

**DOI:** 10.2196/58578

**Published:** 2024-09-23

**Authors:** David Chen, Christian Cao, Robert Kloosterman, Rod Parsa, Srinivas Raman

**Affiliations:** 1 Temerty Faculty of Medicine University of Toronto Toronto, ON Canada; 2 Michael G. DeGroote School of Medicine McMaster University Hamilton, ON Canada; 3 Department of Radiation Oncology University of Toronto Toronto, ON Canada

**Keywords:** artificial intelligence, clinical trial, completion, AI, cross-sectional study, application, intervention, trial design, logistic regression, Europe, clinical, trials testing, health care, informatics, health information

## Abstract

**Background:**

Evaluation of artificial intelligence (AI) tools in clinical trials remains the gold standard for translation into clinical settings. However, design factors associated with successful trial completion and the common reasons for trial failure are unknown.

**Objective:**

This study aims to compare trial design factors of complete and incomplete clinical trials testing AI tools. We conducted a case-control study of complete (n=485) and incomplete (n=51) clinical trials that evaluated AI as an intervention of ClinicalTrials.gov.

**Methods:**

Trial design factors, including area of clinical application, intended use population, and intended role of AI, were extracted. Trials that did not evaluate AI as an intervention and active trials were excluded. The assessed trial design factors related to AI interventions included the domain of clinical application related to organ systems; intended use population for patients or health care providers; and the role of AI for different applications in patient-facing clinical workflows, such as diagnosis, screening, and treatment. In addition, we also assessed general trial design factors including study type, allocation, intervention model, masking, age, sex, funder, continent, length of time, sample size, number of enrollment sites, and study start year. The main outcome was the completion of the clinical trial. Odds ratio (OR) and 95% CI values were calculated for all trial design factors using propensity-matched, multivariable logistic regression.

**Results:**

We queried ClinicalTrials.gov on December 23, 2023, using AI keywords to identify complete and incomplete trials testing AI technologies as a primary intervention, yielding 485 complete and 51 incomplete trials for inclusion in this study. Our nested propensity-matched, case-control results suggest that trials conducted in Europe were significantly associated with trial completion when compared with North American trials (OR 2.85, 95% CI 1.14-7.10; *P*=.03), and the trial sample size was positively associated with trial completion (OR 1.00, 95% CI 1.00-1.00; *P*=.02).

**Conclusions:**

Our case-control study is one of the first to identify trial design factors associated with completion of AI trials and catalog study-reported reasons for AI trial failure. We observed that trial design factors positively associated with trial completion include trials conducted in Europe and sample size. Given the promising clinical use of AI tools in health care, our results suggest that future translational research should prioritize addressing the design factors of AI clinical trials associated with trial incompletion and common reasons for study failure.

## Introduction

The advent of artificial intelligence (AI) is expected to transform the practice and delivery of health care practices, including applications in clinical diagnosis, treatment, and management [1,2]. Adoption of these promising but often untested tools requires systematic evaluation through clinical trials, widely regarded as one of the highest forms of evidence to inform clinical practice [3,4].

It is well known that clinical trials fail to compete at different stages of the research and development process [5,6]. However, the evaluation of AI tools as interventions in clinical trials raises the potential for new modes of trial incompletion that have yet to be explored. Unique challenges in translation AI research can include poor patient cohort selection, ineffective patient monitoring during trials, and logistical difficulties for implementation [7,8].

Prioritizing research to address the common limitations of AI in trials is a critical step toward the validation and adoption of these tools. To address this issue, we performed a case-control study of AI trials in ClinicalTrials.gov to identify trial design factors associated with trial completion and catalog study-reported reasons for trial incompletion.

## Methods

We queried ClinicalTrials.gov, the largest international registry of clinical trials, on December 23, 2023, using AI keywords based on previous methodology to identify AI-related trials (n=6738) [9]. Incomplete trials were defined as terminated, suspended, or withdrawn trials. We only included complete and incomplete trials testing AI technologies as a primary intervention, categorized by complete (n=485) or incomplete status (n=51; Figure S1 in Multimedia Appendix 1). Our study focused on clinical trials that evaluated AI technologies in at least 1 study arm to assess the characteristics of trial completion associated with primary AI interventions. We excluded exact duplicate trials but included separate trials evaluating the same AI intervention for different trial methods or targeted populations. We excluded ongoing trials given their unknown status of trial completion by the date of data collection needed for this study’s case-control design. We excluded observational trials and studies with missing trial design elements such as allocation, intervention model, and masking.

Two reviewers (CC and RK) independently screened all studies for inclusion and data extraction after a pilot on 20 studies to improve interreviewer agreement. Discordance of screening and data extraction were resolved through discussion with a third reviewer (DC) to achieve full agreement across the reviewer team. We extracted trial design factors including the clinical area addressed by AI technology, the intended use population, and the intended role of AI technology (Table S1 in Multimedia Appendix 1). Trial factors with no available data were coded as “Unknown.”

Chi-square tests with Benjamini-Hochberg correction were used to compare the distribution of trial factors between complete and incomplete trials. Logistic regression models were fit for univariable and multivariable analysis of trial factors associated with trial completion. Stepwise variable selection using the Akaike information criterion was used to identify the optimal set of trial factors useful for the multivariable regression model. The propensity score–matched, multivariable regression was conducted using 3 complete trials for each incomplete trial, matched based on all insignificant trial factors from the multivariable analysis. Odds ratio (OR) and 95% CI values were calculated for all factors observed in both complete and incomplete trials. A 2-sided *P* value threshold of .05 was used for statistical significance. This study was completed in accordance with the STROBE (Strengthening the Reporting of Observational Studies in Epidemiology) reporting guidelines [10].

No ethics approval and informed consent were needed since this study analyzed publicly available data and did not include human subjects.

## Results

The majority of AI trials were categorized as being completed (485/536, 90.5%). Trials primarily implemented diagnostic AI interventions (200/536, 37.3%), tested AI interventions intended for health care providers (432/536, 80.6%), and were conducted in adult and older adult populations (397/536, 74.6%). Furthermore, the most prevalent clinical areas addressed in trials included oncology (93/536, 17.4%), cardiovascular system (71/536, 13.2%), and generic health (60/536, 11.2%). Geographically, the majority of trials were conducted in Europe (174/536, 32.5%), North America (145/536, 27.1%), or Asia (142/536, 26.5%), and many trials recruited relatively larger sample sizes (1000 participants; 131/536, 24.4%). We found a paucity of reporting information concerning study allocation (399/536, 74.4% unknown), intervention model (321/536, 60.1% unknown), masking procedures (59.9% unknown), and funding source (438/536, 81.7% unknown) and excluded these factors from the univariable analysis. Multimedia Appendix 2 reports a summary of the included studies.

From the univariable logistic regression model, the role of AI (prediction: OR 3.93, 95% CI 1.32-11.72; *P*<.001), study type (interventional: OR 0.52, 95% CI 0.29-0.92; *P*=.03), continent (Asia: OR 16.0, 95% CI 3.73-68.77; *P*<.001 and Europe: OR 4.11, 95% CI 1.90-9.25; *P*<.001), and sample size (OR, 1.00; 95% CI 1.000-1.003; *P*<.001) were associated with completion of AI trials (Multimedia Appendix 3). All significant factors from univariable analysis were included in our multivariable model. From the multivariable logistic regression model, the role of AI (prediction: OR 4.55, 95% CI 1.44-14.36; *P*=.01), continent (Asia: OR 11.57, 95% CI 2.59-51.73; *P*=.001 and Europe: OR 4.44, 95% CI 1.91-10.3; *P*<.001), and sample size (OR 1.00, 95% CI 1.000232-1.00243; *P*=.02) were associated with completion of AI trials (Multimedia Appendix 3). The case-control, propensity-matched, multivariable logistic regression model found that continent (Europe: OR 2.85, 95% CI 1.14-7.10; *P*=.02) and sample size (OR 1.00, 95% CI 1.000202-1.00218; *P*=.02) were associated with completion of AI trials (Multimedia Appendix 3)

**Figure 1:**
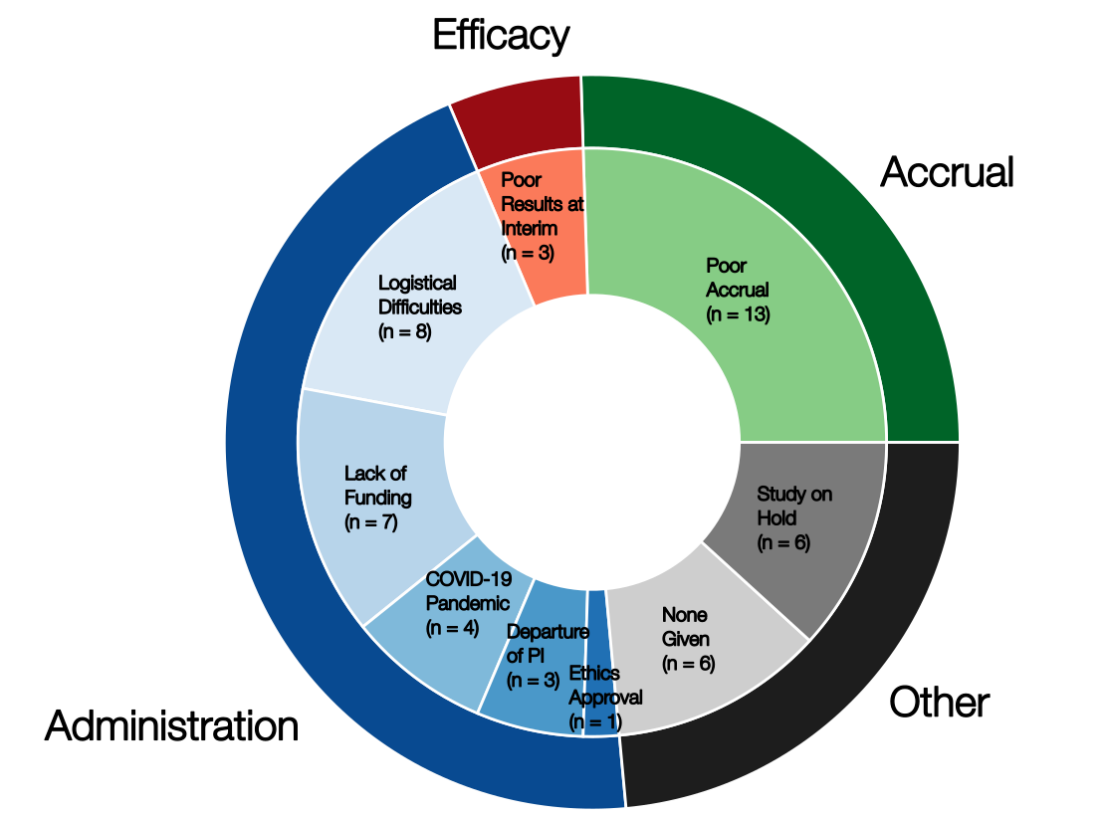
Reasons for failure of clinical trials that evaluated artificial intelligence. PI: principal investigator.

Common study-reported reasons for trial incompletion include poor accrual (13/51, 25.5%), poor results at interim (3/51, 5.9%), administration (22/51, 43.1%), and other (12/51, 23.5%; Figure 1). Among incomplete trials due to poor administration, reported reasons included logistical difficulties (8/22, 36.4%), lack of funding (7/22, 31.8%), COVID-19 pandemic (4/22, 18.2%), departure of investigator (3/22, 13.6%), and lack of ethics approval (1/22, 4.5%).

## Discussion

### Principal Findings

Our multivariable analysis found that clinical trials conducted in Europe were positively associated with trial completion compared with trials in North America. However, we note that trials conducted outside of North America are less likely to be registered on ClinicalTrials.gov [11], which may be due in part to different national and funding mandates, thus potentially resulting in geographic reporting bias. There remains a need for sound methodological design in AI model training to improve generalizability in validation cohorts [12]. We hypothesize that AI models trained on data from local, homogenous cohorts may be more likely to complete but could fail to generalize to external cohorts with increased heterogeneity and a lack of training representation. The design of AI training architectures should consider representation from data-poor sources and due diligence in external validation before clinical implementation [13].

Our findings also highlight the importance of trial design, noting that larger participant sizes correlate with successful trial completion, which may be unique to AI-based trials, where the performance of the tools are enhanced with larger datasets. We note that studies involving large sample sizes may lead to false positive discoveries due to inflation of *P* values [14], and administrative difficulties that can contribute to trial failure [15]. This finding aligns with recent reports on the need to consider appropriate sample size to ensure reliable estimates of AI intervention performance [16] as well as the association between sample size and the sensitivity of detecting differences in study outcomes [17,18]. The emergence of noninferiority trials evaluating the performance of AI interventions compared with standard-of-care controls should require consistent reporting and justification for sample size [19] and should consider the use and challenges of large-scale training and validation cohorts for AI models [20]. Randomized clinical trials (RCTs), including but not limited to trials evaluating AI interventions, should consider sample size with respect to type 1 error, power, effect size of clinical interest, and population variance, as well as justify the use of sample size calculations based on applicable assumptions [21].

Compared with a cross-sectional study of all trials reported in ClinicalTrials.gov [22], our results also demonstrated that administrative reasons made up a greater proportion (45.1% vs 29.8%) of reasons for AI trial failure compared with all trials, and further research is required to understand unique administrative challenges present in AI trials. There remain several key challenges that should be addressed to translate AI interventions in medicine, including the assessment of performance metrics in relation to clinical use, algorithmic biases that limit generalizability to new populations, and logistical difficulties in implementing AI systems into clinical workflows led by clinicians [8]. Broadly, the shift of AI interventions toward integration into compound systems with multiple inputs, outputs, and operators may present new administrative challenges in clinical workflows that should be addressed in future AI clinical trials.

Our study has several limitations. First, this study is limited by the lack of paired trials in the literature that could provide case-control comparisons between different trial design factors. Despite incorporating relevant trial design covariates and applying propensity-matching techniques, simplifying the intricacies of trial completion into its constituent design factors may overlook several other considerations, which could significantly influence trial outcomes. Second, there was a paucity of information reporting, where several study design factors were not consistently reported [23]. Researchers should adhere to reporting guidelines wherever possible to enhance scientific transparency and accountability [24].

### Conclusion

This study suggests that clinical trials that recruited larger sample sizes and conducted in Europe, compared with North America, are associated with successful trial completion. The most common reasons for trial incompletion included poor participant accrual and administrative difficulties. Future research is needed to address the limitations of AI clinical trials associated with trial incompletion to improve the translation of AI into clinical practice.

## Appendix

Multimedia Appendix 1 Inclusion and exclusion criteria and data extraction procedure.

Multimedia Appendix 2 Descriptive statistics of complete and incomplete interventional trials evaluating artificial intelligence.

Multimedia Appendix 3 Predictive statistics of complete and incomplete interventional trials evaluating artificial intelligence.

## Abbreviations

AI: artificial intelligence
OR: odds ratio
RCT: randomized clinical trials
STROBE: Strengthening the Reporting of Observational Studies in Epidemiology

## Data Availability

The datasets generated during and/or analyzed during this study are available from the corresponding author on reasonable request.
