# Supplementary material for: Trial Factors Associated With Completion of Clinical Trials Evaluating AI: Retrospective Case-Control Study
Source: J Med Internet Res. 2024 Sep 23;26:e58578. doi: 10.2196/58578 (PMC11459098; doi:10.2196/58578)
Supplement: Multimedia Appendix 1 [file jmir_v26i1e58578_app1.docx]

**Multimedia Appendix 1**

**Figure S1**. Schematic of clinical trial inclusion and exclusion criteria.

**Table S1**. Dictionary of categorical terms and their definitions for Role of AI in clinical trials.

**Figure S1**. Schematic of clinical trial inclusion and exclusion criteria.


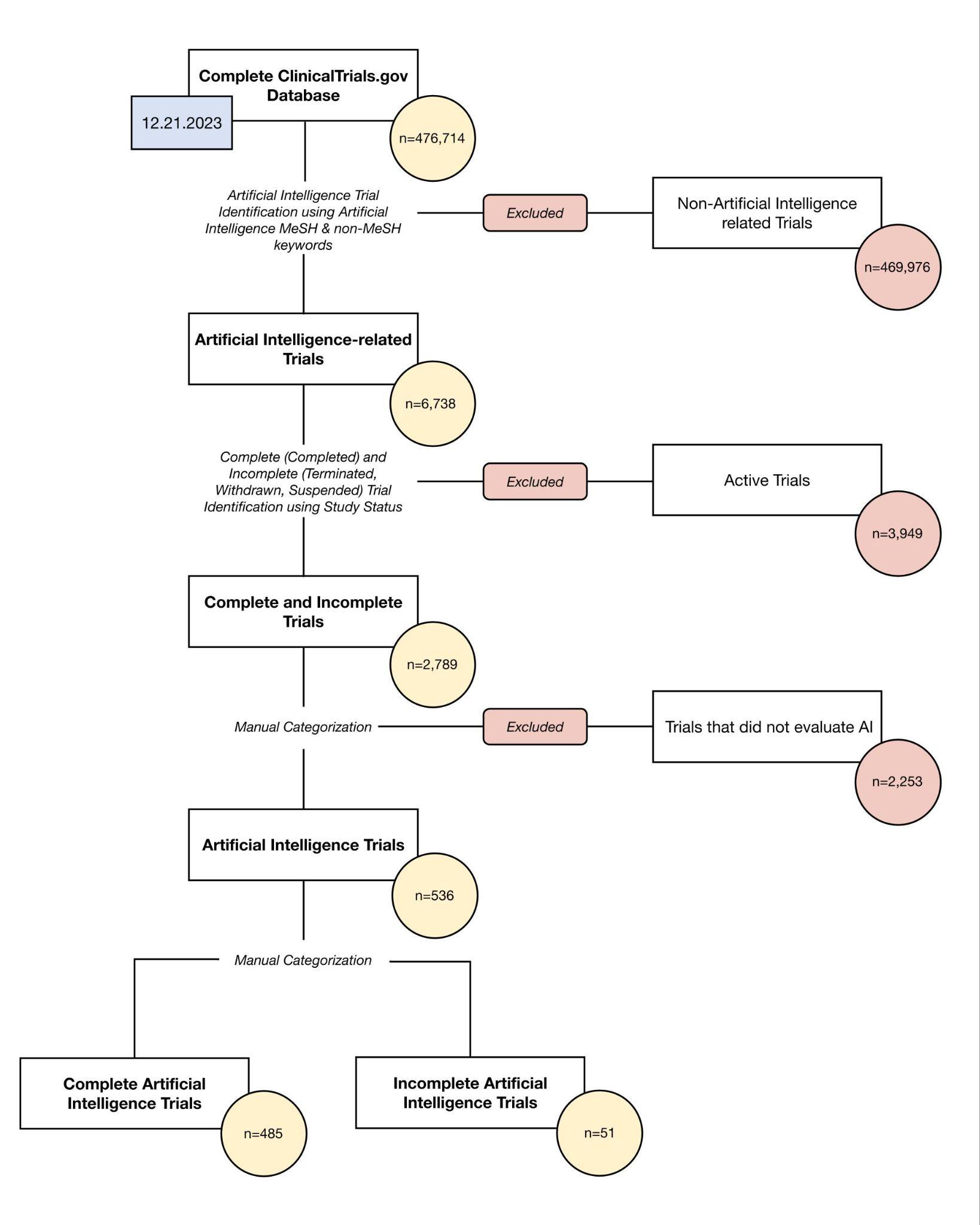


**Table S1**. Dictionary of categorical terms and their definitions for Role of AI in clinical trials.

| **Term** | **Definition** |
| --- | --- |
| Diagnosis | Identification of clinical disease or clinical signs of design |
| Treatment | Active intervention useful for patient care or management |
| Monitoring | Longitudinal monitoring or supportive care of patient over time |
| Screening | Intervention used within the context of a putative clinical screening test |
| Predictive | Prediction of future clinical outcome or clinical risk stratification |
| Other | Trials that could not be assigned to the other Role of AI categories |
